# Macrophage-Induced Pro-Fibrotic Gene Expression in Tubular Cells after Ischemia/Reperfusion Is Paralleled but Not Directly Mediated by C5a/C5aR1 Signaling

**DOI:** 10.3390/life14081031

**Published:** 2024-08-19

**Authors:** Erik Bleich, Eva Vonbrunn, Maike Büttner-Herold, Kerstin Amann, Christoph Daniel

**Affiliations:** Department of Nephropathology, Institute of Pathology, Friedrich-Alexander-University (FAU) Erlangen-Nuremberg, 91054 Erlangen, Germany; erik.bleich@fau.de (E.B.); eva.vonbrunn@uk-erlangen.de (E.V.); maike.buettner-herold@uk-erlangen.de (M.B.-H.); kerstin.amann@uk-erlangen.de (K.A.)

**Keywords:** complement C5a, macrophage/tubular crosstalk, fibrosis, ischemia/reperfusion

## Abstract

Ischemia/reperfusion (I/R) is inevitable during kidney transplantation and causes acute kidney injury (AKI), which affects immediate outcome and leads to chronic changes such as fibrotic remodeling of the graft. We investigated pro-fibrotic signaling after I/R, focusing on the complement component and receptor C5a/C5aR1 and macrophage/tubule crosstalk. Male Dark Agouti rats were subjected to I/R and their kidneys were harvested 10 min, 6 h, 24 h, 3 days, 5 days and 8 weeks after reperfusion. The development of renal fibrosis was assessed by the detection of Vimentin (VIM), α-smooth muscle actin (α-SMA) and collagen by immunohistochemistry and Sirius Red staining, respectively. The characterization of C5a/C5aR1 activity and C5aR1+ cells was performed by multiplex mRNA analysis, ELISA, immunofluorescence flow cytometry and in situ hybridization in animal models and cell culture analyses. In the cell culture experiments, we focused on macrophage/tubule cell crosstalk in co-culture experiments and mimicked in vivo conditions by hypoxia/reoxygenation and supplementation with C5a. Already 6–24 h after the induction of I/R in the rat model, C5a concentration in the plasma was significantly increased compared to the control. The matrix components VIM and α-SMA peaked on day 5 and declined after 8 weeks, when an increase in collagen was detected using Sirius Red. In contrast to early I/R-induced C5a activation, renal *C5ar1* expression was maximal at day 5 and *C5* expression increased until week 8, indicating that the renal upregulation of expression is not required for early complement activation. *C5aR1* mRNA was detected in neutrophils and macrophages, but not in proximal tubular cells in the injured kidneys. The macrophage/tubular cell co-culture experiments showed that macrophages were mainly responsible for the increased expression of fibrosis-associated genes in tubule cells (*ACTA2*, *VIM*, *SNAI1*, *TGFB1* and *FGF-2*), and hypoxia/reoxygenation had a partially enhancing effect. A direct pro-fibrotic effect of C5a was not observed. Increased TGF-ß levels were dependent on the differentiation of macrophages to the M2 subtype. In conclusion, the early activation of mesenchymal markers in tubular epithelial cells leads to long-term fibrotic remodeling characterized by VIM expression and driven by TGF-ß-dependent macrophage/tubular crosstalk. The chemoattractive properties of complement C5a may contribute to the recruitment of pro-fibrotic macrophages.

## 1. Introduction

Ischemia/reperfusion (I/R) injury inevitably occurs during kidney transplantation, especially in deceased donor grafts, and is the most common cause of acute kidney injury (AKI) in renal transplantation [1,2,3]. This type of acute kidney damage can trigger the onset of CKD and increases the long-term morbidity and mortality of transplant recipients [4]. In this setting, CKD mainly refers to chronic transplant nephropathy, which is characterized by interstitial fibrosis with tubular atrophy (IF/TA) and impairs renal function and allograft survival [5,6].

Tubulointerstitial fibrosis is characterized by the excessive production and accumulation of extracellular matrix (ECM) proteins, which are mainly produced by cells of mesenchymal origin, in particular interstitial myofibroblasts, which are characterized by a strong expression of alpha-smooth muscle actin (ACTA2) and Vimentin (VIM) [7,8,9]. Recent studies have shown that the majority of the myofibroblast population originates from pericytes and fibroblasts, but other cell populations such as tubular cells are also capable of contributing to fibrosis development [7]. The complement system plays a crucial role in I/R pathophysiology [1,2]. Its activation leads to direct cell damage not only through the formation of the membrane attack complex, but also the release of anaphylatoxins C3a and C5a, which exert their effects on various cells by binding to their specific receptors C3aR1 and C5aR1 [10]. C5aR1 is expressed in the kidney on cells of the myeloid lineage, such as macrophages and granulocytes, but has also been reported to be present on a variety of non-myeloid cells, such as tubular cells and fibroblasts [11,12,13,14]. While the involvement of the complement cascade in mediating IRI has been extensively studied [1,2], therapeutic approaches to date have not yet shown resounding success, in particular indicating no decisive improvement in long-term graft survival [15,16]. However, the transition from acute IRI to chronic fibrotic remodeling is still rather poorly understood [17]. Therefore, macrophages and complement factor C5a were assessed for their potential role as mediators of early pro-fibrotic changes after IRI in the hope of identifying potential therapeutic targets.

## 2. Materials and Methods

### 2.1. Ischemia/Reperfusion Model in Rats

The protocol for our experimental animal studies was approved by the German regional committee for animal care and use (equivalent to the US IACUC) and authorized by the governmental department (Regierung von Unterfranken, approval number 55.2.2-2532-2-1127). The study was performed in strict accordance with the German Animal Welfare Act. Male WT Dark Agouti rats (150–200 g) were bred and housed under specific pathogen-free conditions in the “Preclinical Experimental Animal Center” of the Medical Faculty of FAU. The surgical interventions and the collection of kidneys at the end of the experiments were performed under isoflurane anesthesia and analgesia using 0.05 mg/kg body weight Buprenovet (Bayer AG, Leverkusen, Germany). The right kidney was removed one week before ischemia/reperfusion and served as a healthy control. Renal ischemia was induced by clamping the arteria renalis for 30 min while keeping the animals at a constant 37 °C using a heatable surgery table equipped with a rectal probe. In total, 48 rats were investigated in groups of 8 animals at 10 min, 6 h, 24 h, 3 days, 5 days and 8 weeks after reperfusion, and kidneys as well as serum samples were collected. To investigate the early events of pathological changes after IRI, we examined time points up to 5 days after injury. In order not to miss a possible early complement activation, we included groups at 10 min, 6 h, and 24 h, because this is when the greatest impairment of renal function is observed. On days 3 and 5, processes can be observed that occur in response to acute kidney injury. In addition, we included a group that was sacrificed 8 weeks after IRI to analyze the long-term consequences of the acute injury.

### 2.2. Immunohistochemical Staining of Rat Kidney Sections

One formalin-fixed paraffin-embedded kidney section per case was stained with Sirius Red according to standard protocol and 4 sections were further processed for immunohistochemistry. The following primary antibodies were used: mouse anti-human α-SMA, mouse anti-human Vimentin (both from DAKO Deutschland, Hamburg, Germany, and cross-reactive against rats), mouse anti-rat CD163 and mouse anti-rat CD68 (clone ED1) (both from Bio-Rad Laboratories GmbH, Feldkirchen, Germany). Subsequently, the sections were incubated with biotinylated secondary horse anti-mouse IgG (Vector Laboratories, Burlingame, CA, USA). Bound antibodies were detected using an ABC Kit and DABImmpact as a chromogen (both from Vector Laboratories, Burlingame, CA, USA) followed by counterstaining of the nuclei using hemalaun. Finally, the slides were digitized using an Axio Scan.Z1 slide-scanner (Zeiss, Oberkochen, Germany). Quantitative evaluation of immunohistochemistry was performed using QuPath version 0.2.3. Software [18].

### 2.3. In Situ Hybridization Combined with Immunofluorescence Staining

For the in situ hybridization of *C5ar1* and *C3ar1*, we used an RNAscope Multiplex Fluorescent v2 Assay Kit and a HybEZ Hybridization System (both from Advanced Cell Diagnostics, Inc., Newark, CA, USA) according to the manufacturer’s standard protocol. The *C5ar1* probes were conjugated with Opal520 and the *C3ar1* probes with Opal620 dyes. After assay completion, the antibodies listed in Supplementary Table S2 were used. The stained slides were covered with VECTASHIELD Vibrance Antifade Mounting Medium (Vector Laboratories, Burlingame, CA, USA) and imaged on a laser scanning confocal microscope (LSM710, Zeiss, Oberkochen, Germany).

### 2.4. Human Cell Culture

All cells were kept at 37 °C, 95% humidity and 5% CO_2_, and used for experiments from passages 5 to 15. Human primary proximal tubular epithelial cells (HPTC) (Cell Biologics Company, Chicago, IL, USA) and the human immortalized proximal epithelial cells HKC-8 and HK-2 (American Type Culture Collection, Manassas, VA, USA) were cultured in dishes coated with 4% collagen I solution (Corning, NC, USA) and containing Renal Epithelial Cell Medium 2 (REM) (PromoCell, Heidelberg, Germany). THP-1 monocytes (American Type Culture Collection, Manassas, VA, USA) were cultivated in RPMI 1640 supplemented with L-glutamine, sodium bicarbonate and 20% FBS (Sigma-Aldrich, St. Louis, MO, USA). All media were supplemented with penicillin (100 IU/mL) and streptomycin (100 mg/mL) (Gibco, Carlsbad, CA, USA).

### 2.5. Differentiation of THP-1 Monocytes to Macrophages

Non-adherent THP-1 cells were incubated with 100 ng/mL Phorbol-12-myristate-13-acetate (PMA) (Calbiochem, San Diego, CA, USA) in RPMI 1640 medium with 10% FCS for 48 h. Differentiation was continued during the next 72 h: an M1-like phenotype using 50 ng/mL IFN-y (Peprotech, Cranbury, NJ, USA) and 50 ng/mL LPS (Invitrogen/Thermo Fisher Scientific, Waltham, MA, USA), and an M2-like phenotype using 25 ng/mL IL-4 (Peprotech, Cranbury, NJ, USA) and 25 ng/mL IL-13 (Peprotech, Cranbury, NJ, USA). PMA-activated THP-1 cells without cytokine treatment were considered M0-like.

### 2.6. Flow Cytometry

Prior to C5aR1 staining, Fc receptor binding was blocked by adding an Fc receptor inhibitory polyclonal antibody (Invitrogen/Thermo Fisher Scientific, Waltham, MA, USA) to human cells and blood samples. The blood samples were received from the Department of Transfusion Medicine, University Clinic Erlangen, after approval by the ethics committee of the University of Erlangen, with the approval number 23-276-Bp. C5aR1 was detected using an Alexa 647 conjugated mouse anti-human C5aR1 antibody (Bio-Rad, Hercules, CA, USA).

After washing them with PBS supplemented with 1% bovine serum albumin (Merck, Darmstadt, Germany), erythrocytes in the blood samples were lysed using BD FACS Lysing solution (BD Bioscience, San Jose, CA, USA). The cells were analyzed in a BD FACSCanto™ II flow cytometer and evaluated using FlowJo_V9 (BD Bioscience, Franklin Lanes, NJ, USA).

### 2.7. Hypoxia and Macrophage/HPTC Co-Culture

**Figure 1:**
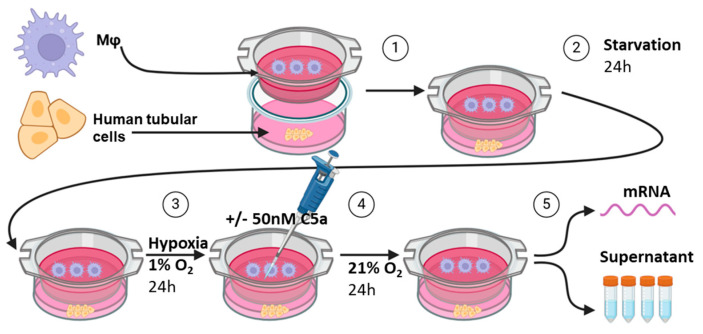
Experimental design of the in vitro co-culture ischemia/reoxygenation experiments. Created with BioRender.com.

HPTC were seeded in 6-well plates and attached overnight. The cells were serum-starved for 24 h and subsequently PET inserts with a 0.4 µm pore size (cellQART^®^, Northeim, Germany) containing M1-like or M2-like macrophages were added to the wells. Immediately thereafter, co-culture and controls without macrophages were cultivated under hypoxic conditions (5% CO_2_ and 1% O_2_) for 24 h, followed by reoxygenation for 24 h with or without the presence of 50 nM recombinant human C5a (Peprotech, Cranbury, NJ, USA). Finally, the cells were collected for mRNA analysis and the supernatants for a TGF-ß ELISA (Figure 1).

### 2.8. Multiplex mRNA Expression Analysis

Fresh frozen rat kidneys were homogenized with a Precellys (VWR International GmbH, Ismaning, Germany) and a QiaShredder (Qiagen, Venlo, The Netherlands). RNA from the tissue lysates and HPTC was isolated using an RNeasy Kit (Qiagen). Concentration and purity were measured with a NanoDrop spectrophotometer (Thermo Fisher Scientific, Waltham, MA, USA). Isolates with a 260/280 absorbance ratio between 2.2 and 1.5 were included. Samples were prepared and hybridized according to the manufacturer’s instructions before gene expression analysis with a NanoString nCounter FLEX Analysis System (NanoString Technologies, Seattle, WA, USA). Custom Codeset panels containing 4 housekeeping genes were used. Quality control and normalization of the data were performed with nSolver Analysis Software Version 3.0 (NanoString Technologies, Seattle, WA, USA), using internal negative and positive controls and housekeeping genes.

### 2.9. TGF-ß ELISA

Cell culture supernatants were analyzed for TGF-ß using a human TGF-ß ELISA kit (R&D Systems, Minneapolis, MN, USA) following the manufacturer’s instructions and using a Synergy microplate reader and Gen5 software (BioTek Instruments GmbH, Friedrichshall, Germany).

### 2.10. C5a ELISA

Rat plasma samples collected at 10 min, 6 h, 24 h, 3 days, 5 days and 8 weeks after I/R were analyzed for C5a using a rat complement component C5a ELISA kit (NBP2-82137, Novus Biologicals, Centennial, CO, USA) following the manufacturer’s instructions and using a Synergy microplate reader and Gen5 software (BioTek Instruments GmbH, Friedrichshall, Germany).

### 2.11. Statistical Evaluation

The data are presented as bar plots with individual values and means ± SD. Cell culture experiments were performed as triplets (C5a stimulation) or quadruplets (hypoxia & co-culture). Normal distribution was determined by the Kolmogorov–Smirnov test and, if applicable, the data were compared to the control using one-way ANOVA followed by Dunnet´s multiple comparison test, or, if not normally distributed, by the Kruskal–Wallis test followed by Dunn’s multiple comparison test. For the comparison of in vitro co-culture experiments multiple comparisons were performed using Tuckey’s multiple comparison test. The correlation of C5a titers with serum urea values was performed using Spearman correlation. For all statistical analyses, GraphPad Prism version 10 (GraphPad Software, La Jolla, CA, USA) was used. *p* < 0.05 was accepted as statistically significant (* *p* < 0.05, ** *p* < 0.01, *** *p* < 0.001, **** *p* < 0.0001).

## 3. Results

### 3.1. I/R Induces Early Pro-Fibrotic Response in Rat Kidneys and Leads to Long-Term Fibrotic Remodeling

**Figure 2:**
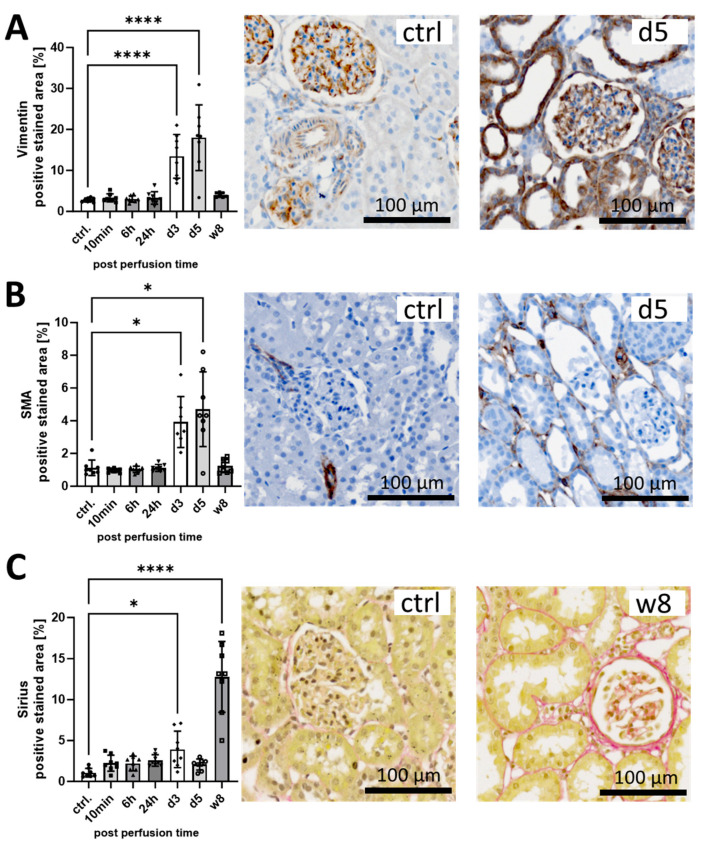
Time course of fibrosis markers in the rat I/R model. The left kidneys were harvested after I/R at the following time points: 10 min, 6 h, 24 h, 3 d, 5 d and 8 w. The healthy right kidney was nephrectomized one week before and served as a control. 2 µm sections of FFPE rat kidneys were utilized and stained for Vimentin (**A**), alpha-smooth muscle actin (**B**) and Sirius (**C**). Subsequently, the slides were imaged on a slide scanner and the positive stained area was detected using manual annotations and a pixel classifier using QuPath Software version 0.2.3. (* *p* < 0.05; **** *p* < 0.0001 vs. ctrl.).

To monitor early pro-fibrotic changes after I/R, we stained for the matrix molecule Vimentin (VIM), the myofibroblast marker α-smooth muscle actin (SMA) and total collagen using immunohistochemistry and Sirius Red, respectively. The positive stained area of VIM (Figure 2A) and α-SMA (Figure 2B) peaked at day 5, reaching 7-fold (VIM) and 5-fold (α-SMA) higher levels, and returned to control levels at week 8. While α-SMA was mainly stained in interstitial cells (Figure 2B), VIM was also clearly detected in tubular structures (Figure 2A). Sirius Red-positive collagen increased only slightly at the early time points but reached a significant 10-fold increase after 8 weeks (Figure 2C). The interstitial Sirius Red staining marks the extracellular matrix deposits at week 8 (Figure 2C). Representative pictures of staining at all time points are shown in Supplementary Figure S1.

### 3.2. Increased Activation of the C5a/C5aR1 Axis Indicates a Possible Pathophysiological Role of C5a in the Rat I/R Model

**Figure 3:**
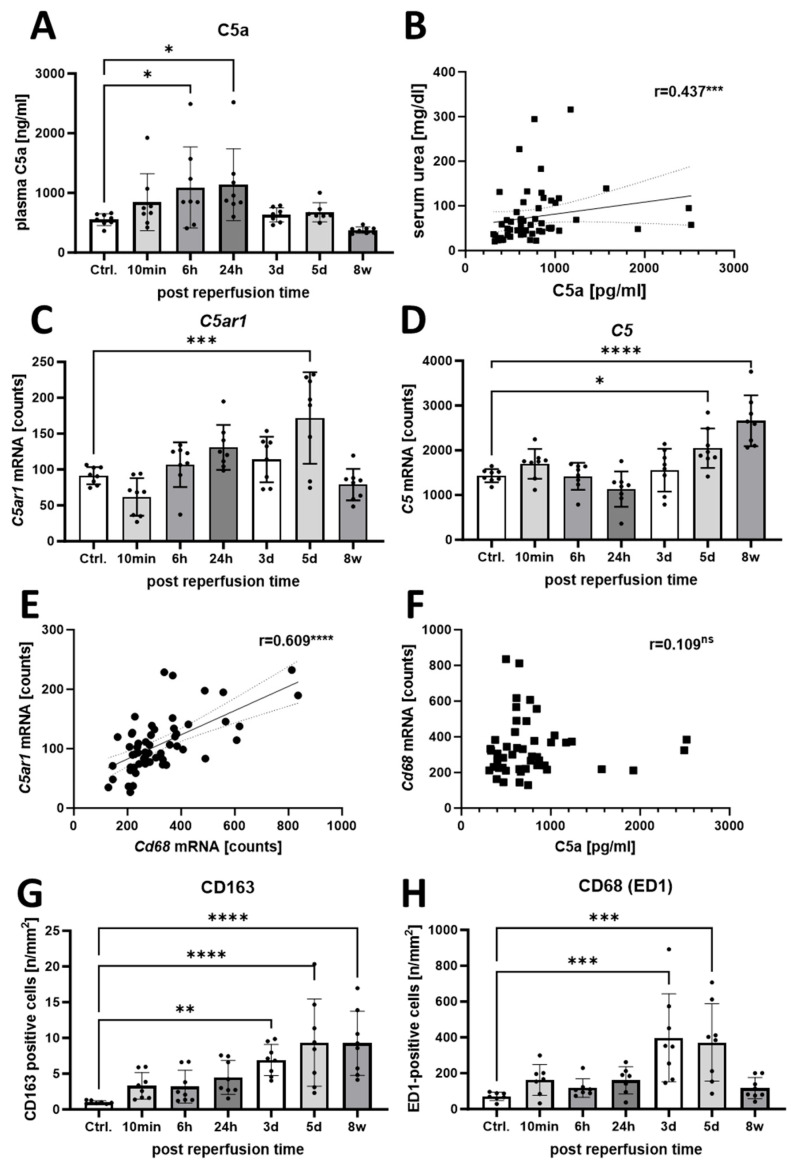
Time course of the C5a/C5aR1 axis, expression in rat kidney sections, C5a concentration in rat plasma and numbers of renal macrophages. The left kidneys were harvested after I/R at the following time points: 10 min, 6 h, 24 h, 3 d, 5 d and 8 w. The healthy right kidney was nephrectomized one week before and served as a control. mRNA was isolated from fresh-frozen rat kidney tissue. C5a was measured in rat plasma using an ELISA (**A**). Plasma C5a concentrations were correlated with serum urea (**B**). Expression of *C5ar1* (**C**) and *C5* (**D**). mRNA was analyzed at different time points post-perfusion. *C5ar1* mRNA expression correlated with the expression of the macrophage marker *Cd68* (**E**), while no correlation was found between *Cd68* expression and plasma C5a levels (**F**). Immunohistochemistry was used for the evaluation of CD163-positive (**G**) and CD68-positive macrophages in kidney sections (**H**) (ns = not significant; * *p* < 0.05, ** *p* < 0.01, *** *p* < 0.001, **** *p* < 0.0001).

To dissect the time course of complement C5a action in the I/R rat model, we detected C5a plasma levels by ELISA and mRNA levels of *C5ar1* and *C5* by multiplex gene expression analysis. Systemic plasma levels of C5a tended to increase after only 10 min, were above control levels after 6 h, and reached twice their concentration after 24 h before falling back to control levels at the later time points (Figure 3A). In addition, C5a plasma levels positively correlated with serum urea levels, a surrogate marker for reduced kidney function (Figure 3B). The mRNA counts of *C5ar1* showed an upward trend at the early time points, doubled the control level at day 5 and dropped again at the last time point after 8 weeks (Figure 3C). *C5* mRNA counts also showed an upward trend after 10 min and 5 days but were highest after 8 weeks (Figure 3D).

*C5aR1* mRNA expression correlated positively with the expression of the macrophage marker *Cd68* (Figure 3E), indicating that *C5ar1* was expressed mainly by macrophages. In contrast, plasma C5a levels did not correlate with Cd68 mRNA expression (Figure 3F). The number of CD163-positive M2-like macrophages increased after the induction of IRI until day 5 and remained at this level for 8 weeks after model induction (Figure 3G). We also observed a similar trend for macrophages stained with the pan-macrophage marker CD68 (ED1), except that the numbers were typically 100-fold higher and had returned to control levels by 8 weeks (Figure 3H).

### 3.3. C5aR1 Is Expressed on CD68+ Macrophages but Not on Tubular Cells in the Rat I/R Model

**Figure 4:**
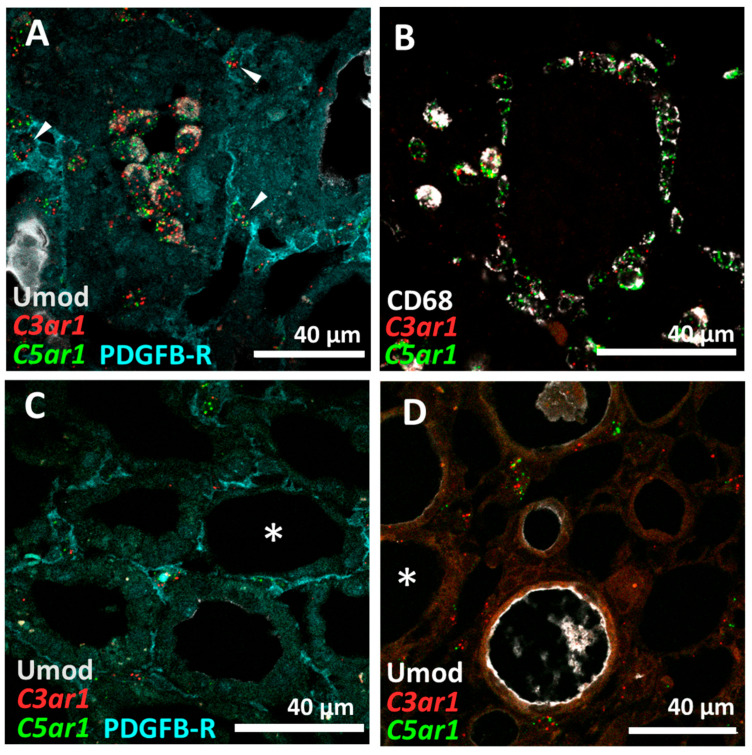
*C5ar1* and *C3ar1* in situ hybridization and immunofluorescence co-localization studies in I/R rat kidney sections. 4 µm sections of FFPE kidneys from d3 post-reperfusion were utilized and processed according to the RNAscope standard protocol. *C5ar1* and *C3ar1* probes were conjugated with fluorochromes, and after assay completion, the sections were incubated with fluorescent antibodies against UMOD (**A**,**C**,**D**) and PDGFRB (**A**,**D**) or against CD68 (**B**).

To verify the cellular targets for C5aR1 signaling, we examined *C5ar1*+ cell populations in the kidney in detail. Since the localization of C5aR1 by immunohistochemistry was inconsistent in previous studies, we localized both *C5ar1* and *C3ar1* by in situ hybridization. Kidney sections were analyzed on days 3 and 5 after I/R as fibrosis markers were significantly elevated at these times. *C5ar1* and *C3ar1* mRNA was predominantly expressed in tubulointerstitial cells (Figure 4A,B and Figure S2) that could be identified as CD68+ macrophages at both time points (Figure 4C,D and Figure S2) and, to a lesser extent, to PDGFRB+ fibroblasts (Figure 4A, arrows), using co-localization studies combining immunofluorescence staining with in situ hybridization. In contrast, significant in situ hybridization *C5ar1* and *C3ar1* signals could not be detected in uromodulin (UMOD)+ thick ascending limb (TAL) nor in other tubular segments lacking UMOD expression (Figure 4C,D, asterisks). Interestingly, most cells with a positive in situ hybridization signal for *C5ar1* also expressed *C3ar1* (Figure 4).

**Figure 5:**
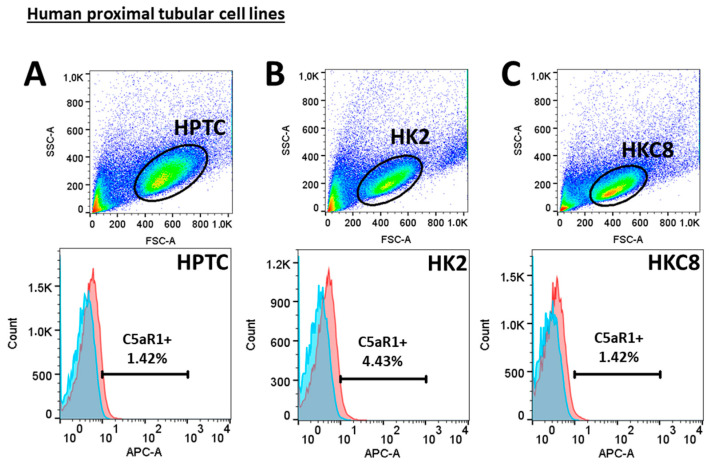
Flow cytometric staining of C5aR1 in proximal tubular cells. Forward–sideward scatter and C5aR1 APC-signal/histogram gated for primary proximal tubular epithelial cells (HPTC) (**A**), HK-2 cell line (**B**) and HKC-8 cell line (**C**). Negative controls were shown as blue and samples as red peaks in the figures below.

**Figure 6:**
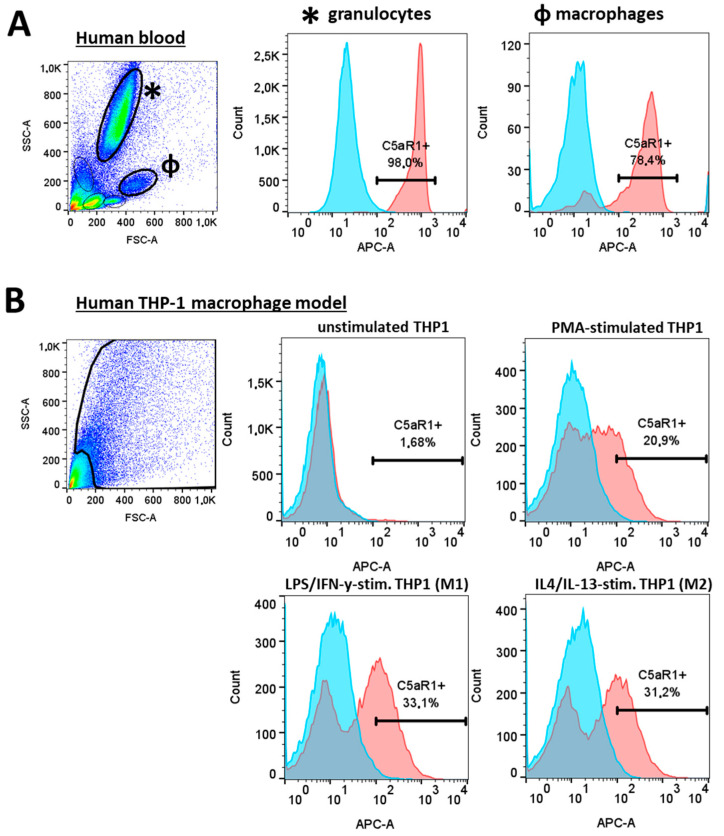
Flow cytometric staining of C5aR1 in human blood and the THP-1 macrophage model. Forward–sideward scatter and C5aR1 APC-signal/histogram of human blood gated for granulocytes and macrophages (**A**). Forward–sideward scatter and C5aR1 APC-signal/histogram gated for macrophages differentiated from human THP-1 monocytes using PMA, IFN-y and LPS (**B**). Negative controls were shown as blue and samples as red peaks in the histograms.

Next, we investigated C5aR1 expression in human cells. Flow cytometry detected C5aR1 expression on HPTC only at a minimal level (<7%), similar to two different tubular cell lines: HK2 and HKC8 (Figure 5). High levels of C5aR1 were found on neutrophil granulocytes (98%) and macrophage cell populations (78%) in human blood samples (Figure 6A). In contrast, unstimulated macrophages generated from a human THP-1 cell line hardly expressed C5aR1 (1.6%) (Figure 6B). After stimulation of the THP-1 cells with PMA to differentiate into macrophages, the percentage of C5aR1-positive cells increased to 20.9%, and further increased after differentiation with LPS and INF-γ into M1 (33.1%) and with IL-4/IL-13 into M2-like macrophages (31.5%) (Figure 6B).

### 3.4. Crosstalk between Human Proximal Tubular Cells and Macrophages Significantly Induced Pro-Fibrotic Response but Was C5a-Independent

**Figure 7:**
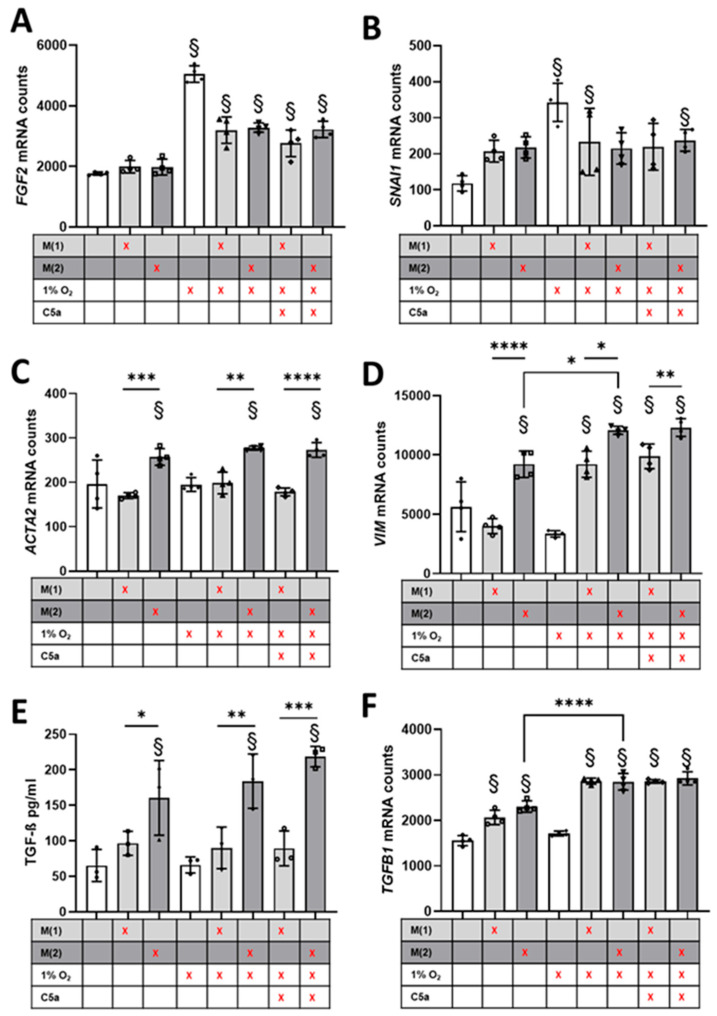
Expression of pro-fibrotic genes in human tubular cells and supernatant concentration of TGF-ß after co-cultivation under normoxic or hypoxic conditions with or without C5a stimulation. mRNA isolated from human proximal tubular cells (HPTC) after a 24 h incubation (21% O_2_ or 1% O_2_) in co-culture with THP-1 monocytes differentiated to macrophages and subsequent stimulation with 50 nM C5a for 24 h. Expression of *FGF2* (**A**), *SNAI1* (**B**), *ACTA2* (**C**), *VIM* (**D**) and *TGFB1* (**F**). Supernatant was collected and TGF-ß concentration was determined by an ELISA (**E**) (* *p* < 0.05, ** *p* < 0.01, *** *p* < 0.001, **** *p* < 0.0001; § *p* < 0.05 vs. Ctrl.).

To investigate the pro-fibrotic interaction between macrophage subtypes and proximal tubular cells after hypoxia in fibrosis, we performed co-culture experiments with human cells. HPTC were co-cultured with THP-1 macrophages differentiated into M1- or M2-like macrophages under hypoxic conditions and subsequently exposed to C5a upon reoxygenation (Figure 1). This allowed us to test our hypothesis that CD68+/C5aR1+ macrophages are potential mediators of C5aR1 signaling and to study the crosstalk between tubular cells and macrophages under normoxic conditions and after a hypoxic event in vitro. We observed a significant hypoxia-dependent induction of *FGF2* (Figure 7A). However, this *FGF2* induction was diminished in proximal tubular cells when co-cultured with macrophages (Figure 7A). *SNAI*, a transcription factor involved in EMT and fibrosis, was also significantly increased by hypoxic stimulation, but co-culture with macrophages alone also led to an increase in *SNAI* expression independent of differentiation, although this did not reach the significance level (Figure 7B). However, we did not observe an additive effect when combining the hypoxia stimulus with the macrophage co-culture, but rather a decrease in hypoxia-stimulated expression as in the expression of *FGF2* (Figure 7B). Macrophage differentiation did not play a significant role for either *FGF2* or *SNAI*. In our experiments, the myofibroblast and early fibrosis marker *ACTA2* was not induced by hypoxia but exclusively by co-culture with M2-like macrophages (Figure 7C). In contrast, *VIM* expression was also affected by hypoxia. Although the hypoxic stimulation of tubular cells alone did not lead to increased expression, it was able to significantly increase expression in M1-like macrophages, whereas M1-like macrophages alone had no effect (Figure 7D). Similar to *ACTA2*, *VIM* expression was significantly increased by co-culture with M2-like macrophages, and in the presence of hypoxia we also observed an additive effect (Figure 7D). The results show that different signaling pathways and matrix components are differentially affected by hypoxia and macrophages. We determined TGF-ß concentrations in the cell culture supernatants as the most important pro-fibrotic cytokine and detected up to 3.4-fold increased TGF-ß levels in co-culture with M2 macrophages, with a tendency towards increasing levels after hypoxia and stimulation with C5a (Figure 7E). Moreover, tubular *TGFB1* gene expression was significantly induced by crosstalk with macrophages (Figure 7F) and further enhanced by hypoxic stimulus. Incubation with C5a during reoxygenation had no significant effect on any of the observed genes (Figure 7A–F). In addition, the HPTC controls did not respond to stimulation with C5a alone (see Source data).

## 4. Discussion

### 4.1. Early Pro-Fibrotic Response in I/R Injury

Maladaptive repair processes after I/R contribute to the progressive fibrotic remodeling of the kidney, culminating in chronic graft failure [4]. By dissecting the time course in our in vivo I/R rat model, we were able to confirm that a pro-fibrotic response is initiated as early as 3 days after I/R, as shown by enhanced tubular staining for VIM and the interstitial staining pattern of α-SMA. VIM indicates structural and functional changes in tubular cells in addition to the induction of α-SMA+ interstitial myofibroblasts at the initiation of repair. These results are consistent with previous studies that have identified tubular *VIM* expression and the interstitial infiltration of α-SMA+ myofibroblasts as early markers of graft fibrosis in human transplant recipients [19,20,21]. Furthermore, in a mouse UUO model, it was recently shown that tubular VIM expression is in part responsible for fibrosis [22]. While repair is critical for graft survival to some extent, it can turn into irreversible fibrotic remodeling in renal transplantation [23], which we were able to observe in our rat model at the final time point of 8 weeks. We hypothesize that this sensitive balance may be tipped towards fibrosis by the interaction of tubular cells with cellular (macrophages and granulocytes) and non-cellular mediators (terminal complement mediators) of the innate immune system.

### 4.2. The Role of C5a in I/R Injury and Fibrosis Induction

In our study, we were interested in the role of C5a in the interaction of macrophages and tubular cells in fibrosis induction. Plasma C5a levels showed a tendency to increase as early as 10 min after the I/R induction and were transiently significantly elevated at 6 and 24 h. This confirms early complement activation over the course of I/R triggered by the primary injury. Despite convincing reports of the pathogenic role of C5a/C5aR1 in I/R and renal fibrosis [2,24,25], the expression of C5aR1 on tubular cells remains somewhat controversial [26]. Based on our findings, proximal tubular cells do not express C5aR1 in sufficient quantity to be a primary target for C5a/C5aR1 signaling. In contrast to the interstitial PDGFRB^+^ fibroblasts and CD68^+^ macrophages, we could not detect distinct in situ mRNA signals for *C5ar1* or *C3ar1* in proximal tubular cells or other tubular cells in vivo in our rat model. Furthermore, we did not detect C5aR1 expression by flow cytometry in primary human proximal tubular cells (HPTC) or in other commonly-used proximal tubular cell lines such as HK-2 and HKC-8. However, other investigators have described the direct stimulation of HK-2 cells with C5a leading to TGF-ß secretion [27], or even the conversion of these cells to an α-SMA+ myofibroblast-like phenotype [28]. Single-cell sequencing data from human kidneys confirm *C5AR1* expression in monocytes/macrophages, while a thick ascending limb cell cluster shows a relatively high mean expression, but no significant fold change compared to all other clusters, indicating high *C5AR1* expression in single tubular cells (Supplementary Figure S3). Similar data are available for mouse kidney cells indicating *C5ar1* expression, especially in inflammatory cells (Supplementary Figure S4). Furthermore, the expression of *C5aR1* mRNA correlated significantly with the expression of *Cd68*, indicating that macrophages are an important target for C5a signaling. The renal expression of *C5ar1* did not reach its maximum until day 5, whereas *C5* expression increased until the endpoint at 8 weeks after model induction, indicating an up-regulation of *C5* and *C5ar1* mRNA in renal cells, or, more likely, an invasion of *C5ar1*-expressing inflammatory cells. The increase in *C5ar1* mRNA in the kidney concurrently peaked with α-SMA and VIM at day 5, as well as increased *C5* mRNA expression at our final time point of 8 weeks, suggesting complement-mediated fibrosis induction. Surprisingly, in our in vitro hypoxia model with macrophages and proximal tubular cells, we found almost no effect of stimulation with C5a, although we were able to confirm the activity of C5a in other assays. While C5a may not directly influence tubular cells nor the expression of the selected genes measured in this study, it could attract high numbers of pro-fibrotic macrophages by chemotaxis, a property of anaphylatoxins that is well established and described in the literature [29].

### 4.3. M2-like Macrophages Increase Pro-Fibrotic Response in Proximal Tubular Cells

The pro-fibrotic potency of the tubular cell-macrophage crosstalk was demonstrated by our in vitro co-culture hypoxia experiments and corroborated by our in vivo observations. From our analysis of HPTC mRNA, we conclude that increased *VIM* expression is not only dependent on a hypoxic stimulus but also on concurrent crosstalk with macrophages. The extent of the contribution of M1- or M2-like macrophages to the pathophysiology of fibrosis is controversially discussed [30], and accordingly we found that both M1- and M2-like macrophages are potential pro-fibrotic crosstalk partners. However, based on our findings, the presence of M2-like macrophages caused the strongest increase in *VIM* expression in tubular cells. In contrast, *ACTA2* (encoding α-SMA), was also induced when tubular cells were co-cultured with M2-like macrophages, but hypoxic induction was lacking in this in vitro setting, indicating a hypoxia-independent stimulation. However, the proportion of M2-like macrophages in our rat I/R model was very low, so it is unclear how much they contribute to fibrosis induction. On the other hand, other M2-like macrophage subpopulations that do not express CD163 are also involved in fibrosis induction [31]. We attributed this outcome to secreted TGF-ß (supernatant) that originates from M2-like macrophages. Our macrophage model comprised this characteristic feature of M2-like macrophages, which has been described before [30,32]. TGF-ß signaling is known to be involved in AKI-to-CKD transition and in the pathogenesis of fibrosis [33]. Furthermore, TGF-ß has been shown to be specifically capable of inducing partial EMT and G2 arrest in tubular epithelial cells [34,35]. SNAI1 (snail family zinc finger 1, known as Snail), a transcription factor whose expression increased significantly in our tubular cells after hypoxia, was shown to be an important factor capable of prolonging TGF-ß-induced G2 cell cycle arrest upon the induction of a pro-fibrotic program in tubular cells [36,37]. This program describes the acquisition of a phenotype with pro-fibrotic secretome by tubular cells without undergoing transition to an ECM-producing myofibroblast [38]. We interpreted the increased expression of *FGF2* and *TGFB1* after hypoxia in tubular cells as a tubular conversion to such a secretory phenotype. We hypothesize that this phenotype could activate fibroblasts and pericytes through paracrine signaling and thus promote the development of ECM-producing myofibroblasts. This hypothesis is consistent with recent findings in mice and human patients showing autophagy-dependent FGF2 production in tubular cells after AKI and the resulting induction of fibrosis [39]. However, unexpectedly, the hypoxia-induced expression of *SNAI* and *FGF2* in simultaneous co-culture with macrophages was diminished. Rather, we expected that the expression of these two genes would be further increased by co-culture with macrophages, since most of the TGF-beta certainly originates from the macrophages. Apparently, the macrophages secrete a factor that reduces, but does not completely prevent, the induction of *SNAI* and *FGF2*. *FGF2* expression remained significantly elevated compared to untreated proximal tubular cells or when co-cultured with macrophages under hypoxic conditions. Co-culture with macrophages in combination with hypoxia obviously influence gene expression in proximal tubular cells. This is shown by the fact that neither co-culture with M1-like macrophages nor hypoxic stimulation induced Vimentin expression, but the combination of both stimuli did. A large number of signaling pathways and matrix molecules are involved in fibrosis. Our results show that some pathways are affected by hypoxia, but others are more TGF-ß-dependent and interact with each other, resulting in a context-dependent up- or down-regulation of the factors involved. Our results show a strong interaction between tubular cells and macrophages after IRI, which may serve as a promising target to prevent chronic transplant nephropathy.

Nonetheless, these results must be interpreted with caution and several limitations should be borne in mind. Fibrosis is a highly complex process, but in our study we analyzed only some of its players. The induction of pro-fibrotic reactions may be stronger in I/R after kidney transplantation, as it has been shown that a previous uni-nephrectomy leads to reduced injury [40]. Our cell culture model is highly simplified, and cells often lose their expression of genes during in vitro culture; therefore, we cannot rule out the possibility that these cells express *C5AR1* in vivo after all, although we did not see this in the in situ hybridization in our rat model. Furthermore, we focused exclusively on the crosstalk of macrophages with proximal tubular cells, while in vivo a variety of cells are likely to be involved (e.g., distal tubular cells and interstitial fibroblasts).

## 5. Conclusions

In conclusion, as key findings of our study, we were able to show that early pro-fibrotic response occurs in proximal tubular cells after I/R that could play an important role in the development of later fibrosis. This response is characterized by high VIM expression and appears to be driven by TGF-ß-dependent crosstalk between macrophages and proximal tubular cells. Since our study provides correlative data but no mechanistic insights into the pro-fibrotic process, future experiments with C5aR1 inhibitors could further elucidate the role of C5a in fibrosis induction after IRI. The targeted inhibition of TGF-ß and further analysis of the secretome of M2 macrophages under hypoxic conditions to identify other important soluble factors as potential new therapeutic targets are possible directions for future research. Early pro-fibrotic events were paralleled by complement activation as shown by increased C5a levels, which did not act directly on proximal tubular cells, but could, for example, attract macrophages by chemotaxis and thus contribute to the activation of tubular epithelial cells and the development of fibrosis. The indirect effects of complement may in part help to explain why complement inhibition alone failed to significantly improve long-term graft survival in clinical studies so far. Further studies to elucidate the complex interactions in fibrogenesis will be needed.

## Data Availability

The source data underlying this article are shared on: DOI: 10.6084/m9.figshare.26527657.

## Supplementary Materials

The following supporting information can be downloaded at: https://doi.org/10.13140/RG.2.2.12329.40807, accessed on 14 July 2024. Figure S1: Representative immunostainings of the time course of fibrosis markers in the rat I/R model; Figure S2: C5aR1and C3ar1 in-situ-hybridization comparison d3 and d5; Figure S3: C5AR1 expression comparison across clusters in human kidney samples; Figure S4: C5AR1 expression comparison across cell groups in adult mouse kidney samples. Reference [41] are cited in the Supplementary Materials.
